# Comparative Anatomy

**Published:** 1838-04-01

**Authors:** 


### COMPARATIVE ANATOMY.

### I. NERVOUS SYSTEM OF THE AMPHIBIA.

I. ILLUSTRATIONS OF THE COMPARATIVE ANATOMY OF THE NERVOUS SYSTEM. By *Joseph Swan*. Part III. Price 7s. Quarto, pp. 21. Plates VI. Longman's, London, 1837.

II. OUTLINES OF COMPARATIVE ANATOMY. By *Robert E. Grant*, M.D. &c. &c. &c. Part Third. London. Bailliere, 1836. Part Fourth, 1837. Price Four Shillings each Part.

MR. SWAN is, as all our readers, nay as all the profession know, a very zealous cultivator of anatomy. With unwearied assiduity, he ascertains the facts on which hypotheses are grounded and by which they must finally be tried. To his pages we may always turn with the certainty of finding faithful representations of nature, and the library of the philosophical physician or surgeon is defective unless it contains them.

The first Plate of the present Part exhibits the distribution of the olfactory nerve, of the fifth nerve, of the splanchnic and visceral branches of the sympathetic nerve of the turtle.

The second plate represents the sympathetic nerve of the turtle.

The third plate gives a general view of the nerves of the turtle.

The fourth plate represents the brain of the turtle—the nerves of the frog—the brain and posterior or superior surface of the spinal marrow of the same—the brain of the boa-constrictor—the spinal cord of the same.

The fifth plate displays the origin of the cerebral nerves of the boa-constrictor.

The sixth plate displays the sympathetic nerve of the snake.

The part concludes with some observations on the nervous system of the amphibia, which we shall introduce to our readers. We should premise that these observations have been entirely derived from dissections of the snake, the frog, and two species of turtle, a circumstance which Mr. Swan does well to mention in a distinct manner.

The brain of amphibia, says Mr. Swan, differs from that of fishes externally, in the size and shape of several parts. In the larger size of the anterior lobes, in which there is a capacious ventricle, a prominence in this that may be compared with the striated body and a choroid plexus, in not having any mammillary eminences. In having small thalami, connected together in the turtle by a very tenacious commissure, but not placed as in mammalia with respect to the lateral ventricles, but behind these and out of the cavities, and their situation is even more remarkable in the frog. In having posterior as well as anterior commissures, the anterior alone existing in some fishes. The ventricle in the optic lobe is very similar; but in the floor of this, the logitudinal bands extending towards the calamus scriptorius are more prominent. It differs in the very dense membrane covering it, especially in the turtle, in not having so much space in the skull, and not being placed in a fine reticulated membrane containing fluid. In having a pineal gland as well as pituitary one.

The cerebellum of the turtle differs from that of fishes in its smoothness and roundness, in not having lobes or an appearance of convolutions, as in the skate; in the thinness of its parietes, and the greater capacity of the ventricle.

In the snake, the brain is nearly the same as in the turtle; and there is not much difference in the frog, except that the thalami appear externally. In the snake and frog the cerebellum is so small, as hardly to bear a comparison with that of the turtle or fishes.

Dr. Grant remarks that;—in the perennibranchiate amphibia, and in the larva state of those which lose the gills, the spinal cord, the medulla oblongata, and the cerebral parts contained within the cranium present the same proportions and general conditions which we observe as permanent characters in most of the osseous fishes; but the cerebellum is generally smaller in amphibia and reptiles than in all the other vertebrata. As in the lower fishes, the spinal chord in these inferior forms of amphibia is prolonged, small and tapering, through the greater part of the coccygeal vertebræ, without distinct enlargements where the nerves usually come off to the arms and to the legs. The medulla oblongata is yet broad and lobed, the cerebellum in form of a very small median transverse lobe without hemispheres, the optic lobes large, cineritious, smooth without, hollow within, and quite exposed, and the cerebral hemispheres, extended longitudinally, smooth and cineritious externally, without internal ventricles, and smaller than the optic lobes. The metamorphosis of the caducibranchiate species changes the condition of their nervous system from that of the lower fishes to nearly that of the reptiles above them; and these changes are effected so rapidly that we can perceive a marked advancement in the development of the nervous system of the tadpole produced in one day.

The long narrow cerebral hemispheres of adult frogs, proceeds Dr. Grant, taper to the olfactory nerves which commence with cineritious tubercles, and the optic nerves are observed distinctly to cross each other before the optic tubercles. The changes effected in the nervous system by the metamorphosis of the higher amphibia closely resemble those produced by development in the human embryo.

In the class of reptiles, we still quote Dr. Grant, the small cavity of the cranium nearly corresponds with the dimensions of the enclosed brain, as in some fishes, and the superficial cineritious substance still predominates over the internal white fibrous matter, though to a less extent than in fishes and amphibia. The cerebellum is remarkable for its proportionate smallness in this class, and the cerebral hemispheres, containing each a distinct ventricle, now always exceed the optic lobes. The spinal chord of serpents, from their want of arms and legs, is still destitute of enlargements, as in the apodal fishes, but the medulla oblongata is of considerable size, and the fourth ventricle, still uncovered by the small cerebellum, descends into the spinal chord. From the smallness of the brain and cranial cavity in the centre of the head of reptiles, the relative size of these parts does not influence that of the whole head at different periods of life, and the head preserves the same proportions to the rest of the body through life also in amphibia and fishes. From the still imperfect development of the cerebral parts in this class, the vital functions of reptiles are less immediately dependent on them than in hot-blooded animals, and they longer survive their mutilation.

We now return to Mr. Swan. The observations we are about to introduce appear to us to be important, particularly at the present moment, when the excito-motory hypothesis is attracting general attention.

"The brain of amphibia, compared with that of fishes, is rather more complicated, but still has very little development of parts ministering to the general functions of the body; the principal portions are larger or smaller, according to the required extent of the senses and the power of intellect. In both these classes the oblong medulla bears a greater proportion to the size of the brain than in the higher, and seems to be equally connected with the animal functions, and the lobes of the brain, as in them, to be superadded. The large size of the spinal marrow, as compared with that of the brain, is also very remarkable. When, therefore, the vast intricacy and number of the organs of the body in both these classes is considered, and that they are nearly as great as in the two higher classes, and when it is observed how small the size of the brain is in proportion to the nerves, it must be concluded that both the oblong and spinal medulla have peculiar and important functions; as the nerves bear no proportion to the size of the brain, but only to that of the oblong and spinal medulla, it must also be concluded that their power is principally derived from these parts. Appropriate functions are, however, performed by the nerves and their ganglia, and it may be presumed that some power is inherent in them, for there is very little else belonging to the nervous system of many invertebrated animals.

The power of the nervous system is, in many respects, increased or diminished according to the quantity of nervous matter forming the centres, and the supply and quality of the circulating fluids. When the blood is less perfectly changed by respiration, the brain is less, as in fishes and amphibia; the spinal marrow, however, is not less, but is proportionate to the requisite quantity of nerves for the supply of the body, as in birds and mammalia. The more extensive change of fluids, as in these higher classes, is not therefore necessary for the preservation of the nervous matter; but a greater bulk of brain under this imperfect oxygenization of the blood, as in fishes and amphibia, is not generally consistent with the free and healthy functions of the various organs of the body, and even with the maintenance of life. The structure of the brain and spinal marrow of fishes and amphibia is somewhat different from that of birds and mammalia; but they can display great muscular power in the same manner, although it is doubtful whether they can maintain it for the same time.

When the circulation is more languid, the animals exposed to low temperatures, and the motions of the tail not very great in individual parts, it appears that the continuation of the spinal marrow is preferred to the usual cauda equina of mammalia; and it is very probable that the power of the nerves is thus less easily intercepted, and the functions of the parts, under all circumstances, are more certainly preserved. But even in the higher classes, and particularly in birds, when the spinal marrow can be conveniently accommodated, it may with greater security allow of the distribution of the numerous small nerves issuing from it." 71.

Of course it would be unphilosophical to look upon the condition of the nervous system or on that of the vasculo-respiratory as respectively cause or effect—the one of the other. The entire class to which the animal belongs holds a definite rank in the zoological scale, inferior to one, superior to another. The subordinate systems of which it is composed harmonize with one another, and are all regulated by some general laws and occult formative power. If here and there some particular organ is more highly developed than in higher classes, it is evidently for a special purpose, and even its perfection is confined within definite limits.

Dr. Grant remarks that in the saurian and chelonian reptiles the posterior and middle enlargements are obvious on the spinal chord at the origins of the nerves of the extremities. The wide medulla oblongata within the cranium is marked longitudinally by the limits of its three component fasciculi on each side, and the decussating bands of its corpora pyramidalia are more distinct than in fishes. The nerves of the body bear a large proportion to the size of the cerebral centres, and correspond in their distribution to those of the higher air-breathing classes. The great ganglia and plexuses of the sympathetics now more closely accompany the arterial trunks, as we see to become more exclusively their distribution from the articulated and the molluscous classes up to man. The twelve pairs of cranial nerves are seen here as in birds and mammalia, and they chiefly arise from the enlarged medulla oblongata.

The following statements of Mr. Swan are full of anatomical interest.

"In amphibia the nerves are firmer than in fishes, and acquire much magnitude from the addition of neurilema. In the snake, those about the throat are not only tortuous, but in a considerable degree elastic, and thus allow of being sufficiently extended in deglutition. They are large in comparison with the whole brain, but accord with the size of the oblong and spinal medulla, as in fishes. In the turtle and snake, the olfactory nerves have not distinct ganglia: after being joined by the first trunk of the fifth, they are distributed in coarse branches on the Schneiderian membrane, and appear similar, although the turtle feeds on vegetable, and the snake on animal substances; but it may be remarked, that their structure corresponds very much with that of the splanchnic plexuses, which have not ganglia intermixed with, or giving immediate origin to them. The origin of the optic nerves is from the optic lobes and thalami, and not from the optic lobes and mammillary eminences, as in fishes. In the other nerves of the orbit there is not any particular change, except that the sixth is larger, and supplies the additional muscles, which do not exist in fishes; and in the turtle the ciliary nerves proceed from a junction of branches from the third and fifth nerves, but this has not the form of a ganglion. The fifth has not a very distinct smaller portion for joining the third trunk, but there is a greater resemblance to the form of the Gasserian ganglion than in fishes; the usual cutaneous branches pass through perforations in the bone to the parts beneath the horny covering in the turtle, but this disposition does not exist in the same degree in the snake, and is therefore a peculiarity for corresponding with the conformation of the parts receiving them. The par vagum and glosso-pharyngeal at their origins are not very different from the same in fishes, except that they are not so much connected with the auditory nerve; but even in the turtle and snake there are differences which may be best judged of by a reference to the plate. In the snake so many communications between the glosso-pharyngeal, par vagum, and ninth, take place before branches are given off to supply any parts, that it is very difficult to determine with precision the destination of each, for the muscles and other structures of the throat are so complicated, and at the same time so connected in function, that although the nerves be different at their origins, yet it seems they must be connected for associating the actions of the parts receiving them. In amphibia the par vagum appears to supply almost entirely the heart. When the slight difference between the summit and base of the brain in the turtle and skate is considered, it cannot fail of being remarked that so many more nerves exist in the turtle than in the skate, as the hard portion of the seventh, a more distinct auditory nerve, the ninth and accessory; the brain of the skate is, however, more simple in some respects, and several parts of the body are different from the turtle." 72.

Mr. Swan states that, with respect to the sympathetic nerve, there are several varieties in different genera of amphibia. In the turtle and snake, there is not a canal in which the sympathetic passes with a vertebral artery; the branch, according with the one ascending from the first thoracic ganglion in mammalia, passes on the outside of the spine in the turtle, and in the snake, in an incomplete canal near the connexion of the ribs with the eleven superior vertebræ. In the turtle, there is a prolongation, but no cervical ganglia; it is more or less closely attached to the par vagum, but does not exist in the snake, or, if it does, its small size prevents its being easily recognised and distinctly separated. The sympathetic does not appear to be more highly evolved than in fishes; for in the turtle, the ganglia attached to the prolongation are very diminutive, except the first thoracic, which is broad, and has a membranous character; and in the snake, except at its superior part, where there is one remarkable ganglion connected with the trunk of the par vagum, and several smaller ones at the junction of the sympathetic with branches of the second trunk of the fifth proceeding to the palate and nose; in the snake for some distance there are very slight plexiform junctions of the branches from the spinal nerves which join the prolongation; but these plexuses are in some parts more complicated, and in both the snake and turtle exist for giving off the nerves to the viscera instead of the close and fleshy semilunar ganglia. In the frog as in the snake, except the first cervical, no other distinct ganglia of the sympathetic exist, and the branches destined for the abdominal viscera also join a prolongation from which the splanchnic branches are derived.

If any thing definite is to be determined with respect to the functions of the sympathetic, it will be mainly effected by tracing its development through the ascending series of animals. A dependence of the cerebro-spinal system, it will perhaps be possible, with the advance of knowledge, to trace its advance from a simple spino-cerebral plexus to a system such as we see it in man, with its special ganglia and its special apparatus of nerves and plexuses.

The last observations of Mr. Swan relate to the spinal nerves. In the snake, he tells us, they are not very different from those of fishes, except in the closeness of their origins, and the more regular communications of the anterior and posterior bundles of each nerve, and having more distinct and fleshy ganglia. In the frog there is one large nerve instead of a number forming the axillary plexus, as in the turtle; in both however there is a plexus resembling that in the higher classes for giving off the nerves of the posterior extremities, and the spinal nerves, entering it, correspond with these, inasmuch as they have their origin from the inferior part of the spinal chord, just above that from which the caudal nerves usually issue.

We cannot take our leave of Mr. Swan without strongly recommending his work to the notice of our readers, nor without urging himself to persevere in his honorable and scientific labours. Those labours are of the best description, because they consist in the determination of particulars, on which all generalizations must be based. But we would venture to suggest to Mr. Swan, for the sake of the reading public, as well as for his own, to add to those particulars, generalizations and summaries as wide and comprehensive as exact knowledge will permit. His researches being thus rendered more pleasing will in the same ratio be more instructive.

### II. Comparative Anatomy of the Digestive Organs, Chyliferous and Sanguiferous Systems.

The two parts of Dr. Grant's Outlines before us present a very fair account of the apparatus of digestion in the various classes of animals. It is traced from its most simple to its most complicated forms and pursued through its various phases of development.

We shall not attempt a complete account of so extensive a subject, an account which must necessarily comprise details unsuited for a practical journal. We shall merely present in a series of slight sketches, a notice of some of the more striking circumstances connected with the comparative anatomy of the digestive organs.

Dr. Grant commences their examination with some general observations of an interesting character. We extract them.

"As an animal," he says, "is but a moving sac, organized to convert foreign matter into its own likeness, all the complex *organs of relation* or *of animal life* serve but to administer to this digestive bag. The *bones*, connected together by their *ligaments*, are but the solid levers which enable the *muscles* to move it to and fro, and the *nervous system*, with its various *organs of sense*, serve but to direct its movements in quest of food. The unorganized food of plants is placed by nature in contact with the exterior surface of their body, and their vessels are directed thither to select and absorb it, which roots them through life to the soil where they grow ; but as animals place their food within their stomach and have their roots directed inwards to that central reservoir, they can change their place and move about in quest of what is most congenial to their nature. The *organs* of *animal life* relate to this difference between the two organic kingdoms, to the locomotive powers of animals for the selection of their food ; but the *organs* of *vegetative life* relate merely to the assimilation of food when already within the body, and are, therefore, common to animals with plants. The alimentary surface of the plant is the exterior of its root, ramified and fixed in the soil which affords it food, so that a vegetable is like an animal with its stomach turned inside out. As the organs of relation are those most immediately connected with the varying external circumstances of animals, they are the most variable in their character and inconsistent in their existence ; but those of vegetative or organic life relating to the more common and necessary functions of assimilation are much more regular and constant in their character. No organ, indeed, is more universal or essential in animal bodies than that internal digestive cavity by which they differ so remarkably from the species of the vegetable kingdom. This internal sac is but an extension of the primitive absorbent surface of the skin, which, in animals, passes into or through the homogeneous cellular tissue of the body. And, although in the simplest forms of animals, this primitive sac performs alone all the assimilative functions, we find it, as we ascend in the scale, giving origin to various other organic systems, to which distinct parts of the complex function of assimilation are successively entrusted. Thus the peripheral nutrition of the plant passes gradually into the central mode of the animal, and the organs of organic or vegetative life, whether they open internally into the digestive cavity, or on the mucous surface of the skin, may be considered as originating from the exterior integument, which is itself only a portion of the primitive cellular tissue of the body, modified by the stimulating contact of the surrounding element. As the various tubulent prolongations of the alimentary canal become more and more developed and isolated from their primitive source, they assume properties and functions more and more peculiar and distinct, and thus form the numerous follicular and conglomerate glandular apparatus, and the various vascular systems, of animals. An alimentary cavity is observed in every class of animals and almost in every species, and its form and structure vary according to the situation of animals in the scale, or according to the kind of food on which they are destined to subsist, and the extent of elaboration it requires to undergo to assimilate it to the animal's body. The peculiarities presented by the digestive organs are, therefore, intimately connected with the diversities of form manifested by the organs of animal life, and with all the living habits and instincts of animals." 305.

### A. DIGESTIVE ORGANS OF THE CYCLO-NEUROSE, OR RADIATED CLASSES.

The lowest class of animals is termed by Dr. Grant the cyclo-neurose, or radiated, from the anatomical disposition of its nervous system. In this class Dr. Grant examines the digestive organs as they present themselves in the polygastrica—the poriphera—the polypiphera—the acalepha—the echinoderma. We shall not follow strictly this scholastic arrangement.

A. *Polygastrica.* In the cyclo-neurose class, we find the simplest forms of animal existence. In reference to the digestive apparatus, perhaps, the polygastrica can hardly be considered to present the *most* simple. *They* derive their name from the numerous internal digestive cavities observed in them. Dr. Grant gives a general account of them in these terms:—

From the great transparency of these microscopic animals, their digestive sacs, when empty, or when filled only with water as they often are, appear like portions of the common cellular substance of the body, or like gemmules or internal animalcules, and from not being easily or generally recognised as alimentary cavities, many, like Lamarck, were led to believe that these animals were without a mouth or any internal organs, and were nourished by superficial absorption, like marine plants. Lewenhoeck, however, observed that they possessed an internal cavity, and devoured each other; the same was seen by Ellis; Spallanzani perceived them swallowing each other so voraciously that their bodies became distended with their prey; and Goetze designated the *trichoda cimex* the wolf of infusions from its rapacity among the smaller animalcules. Gleichen placed animalcules in infusions coloured with carmine in order to discover the forms of their digestive cavities, and he has figured many *trichodæ, vorticellæ,* and other animalcules with their internal sacs filled with this coloured matter; the same was done by Trembley; and these stomachs are figured by Müller, Bruguiere, and most others; but Müller supposed that they fed upon water, from their stomachs being most frequently filled with that fluid. Ehrenberg has more extensively employed opaque colouring matter to detect the forms of these internal cavities, and by using principally carmine, sap-green, and indigo, carefully freed from all impurities which might prevent their being swallowed, he has succeeded better than his predecessors in unfolding the structure of the digestive organs of animalcules. Such coloured organic matter, diffused as fine particles mechanically suspended in the water in which animalcules are placed, is readily swallowed by them, and renders visible, through their transparent bodies, the form and disposition of their alimentary cavities; but, however long they remain in these coloured infusions with their stomachs distended with the colouring matter, it is not perceived to communicate the slightest tinge to the general cellular tissue of their body. They appear to possess an acute sense of taste in rejecting coloured metallic and other substances which might prove hurtful to them, and their food appears to consist chiefly of smaller animals of the same class and of particles of mucus or other decomposed organic matter found in the water.

In the simplest of the polygastrica, there is but one general orifice to the alimentary cavities, which is placed at the anterior extremity of the body and is surrounded with long vibratile cilia, which serve both as organs of motion and tentacula. The several stomachs covered by the general wide integument, open by distinct short œsophageal canals into the common buccal orifice, and there is no separate anal aperture for the excrementitious residue of digestion. In the *monas termo*, continues Dr. Grant, which is only about the two-thousandth of a line in diameter, four to six of these small round stomachs have been observed filled with colouring matter, although they did not appear to be half the number which might be contained in its body; each of these stomachs, of about the six-thousandth of a line in diameter, appears to open, as in other *anentera*, by a narrow neck into a wide funnel-shaped mouth, surrounded by a single row of long vibratile cilia, which attract the floating organic particles, or minuter invisible animalcules, as food.

B. *Polypiphera.* The hydra, or fresh-water polype presents a very simple form of digestive apparatus. It has been described particularly, as our readers must be well aware, by Trembley. The polype exhibits a simple sac excavated in the cellular substance of the body, and destitute of all cœca or glandular appendices, and even of a distinct anus. The parietes of this simple polype appear to possess the same properties in every part, as they continue to seize and digest food when the animal is turned inside out, and each part of the animal, when cut to pieces, is found to develop itself to a perfect polype. What was formerly the internal digestive surface is found also to become the generative, and to produce gemmules and young polypes when the animal is turned inside out. They feed chiefly on larvæ and annelides which they search for and seize by the long tentacula developed from the sides of their mouth, and they often swallow animals many times larger than their own body, by stretching their thin elastic parietes over their prey. The digested part of the food passes through the common cellular tissue of their body, and through their tubular tentacula, and the residue is thrown out by the mouth.

C. *Poriphera.* Another very simple form of digestive apparatus is observable here. It approaches very nearly to that of plants.

The cellular tissue of their body is permeated in all directions by ramifying and anastomosing canals, which begin by minute superficial pores closely distributed over every part, and terminate in larger orifices variously placed according to the exterior form of the entire animal. In the minute superficial absorbent pores we can generally perceive a fine transverse gelatinous net-work and projecting spicula, to protect these entrances from the larger animalcules and from noxious particles floating in the water. The internal canals, like the veinous system, leading from capillaries to trunks, are bounded internally by a more condensed or more highly organised portion of the general cellular substance of the body, and are incessantly traversed by streams of water, passing inwards through the minute pores, and discharged through the larger orifices or vents; but no polypi have been observed in any of those parts, nor even cilia, although from analogy we may suppose them necessary as the active agents of the currents. In this simple organization there appears to be only an increased extent given to the general cutaneous absorbent surface; there are yet no distinct cœca or stomachs for receiving and retaining the aliment that has been conveyed into the body along with the currents of water. From the incessant manner in which these traverse the body, it would appear that all parts of the interior of these animals, like the exterior surface, of their general cellular tissue, are adapted to admit by endosmose, and to assimilate nutritious matter to the texture of their body. On watching the streams of water that issue from the vents, minute flocculent particles are observed incessantly detached from the interior, and thrown out with the currents, these appear to be fine mucous pellicles excreted from the surface of the internal canals, as the residue of digestion thus detached from the body. A similar mode of excretion is often seen on the naked mucous surface of zoophytes, where thin pellicles are periodically detached from the soft exterior of the body.

So far as we have gone the digestive apparatus has been limited to this:— either it has been one large pouch, with a single aperture armed with instruments of prehension, as in the hydra—or a similar pouch with cœca developed from it, as in the polygastrica—or a canal, with an orifice of ingress and of egress, and a current of water, carrying the nutritive matter, passing through it. A little higher and we perceive the simple idea represented by these several contrivances carried farther.

D. *Acalepha.* We see the central cavity provided with several canals or apertures of ingress. The resemblance to the spreading roots of trees has obtained for the creature which offers an apparatus of this sort, the name of rhizostoma,* from the immortal Cuvier. Such animals, like inverted zoophytes torn from their fixed attachment and floating through the sea with their polypi extended in all directions, have numerous small pendent orifices at the extremities of peduncles more or less ramified and extended, and these polypiform mouths lead by narrow canals to a central sac, from which the nutritious matter is sent by numerous radiating ramified ducts to all parts of the body. Larger and more direct openings, varying in number in different animals, are also generally observed leading into this gastric cavity, which is sometimes central and single, and in others is divided into compartments disposed around the vertical axis of the disk.

E. It is obvious that the digestive cavity would be perfected by having a distinct aperture for ingress, and another for egress, at its extremities. We should then have a digestive canal receiving the nutriment at one end—retaining it for a sufficient time—and ejecting at the other end the useless residue. In other words, we should have a mouth and an anus. We see this in many of the polygastrica. There is in many of them an alimentary canal, with an oral and an anal orifice, which traverses the body, and is provided with numerous small round cœcal appendices, which open into its parietes throughout its whole course, and which appear to perform the office of stomachs in receiving and preparing the food.

A similar arrangement obtains with some of the acalepha. The Beroë pileus has a straight alimentary canal passing through the long axis of its body, commencing at the lower part with four thin prominent contractile and highly irritable lips surrounding the wide oral aperture. The contracted œsophageal part is succeeded by a gastric expansion of this straight canal, containing frequently minute entomostracous crustacea which have been swallowed as food, and a narrow straight intestine terminates in a prominent anal orifice at the upper part of the body.

In the *Echinoderma* this disposition becomes more general, and we see in many of them the evident preparation for the arrangement which becomes common in the *Articulata.* To the latter therefore we pass.

### B. Digestive Organs of the Articulata.

A. The *Entozoa* present generally the simplest form of alimentary apparatus met with in the articulata. In all the nematoid and more perfect forms of entozoa the alimentary canal passes simple through the body, presenting a distinct oral and anal aperture which are generally at the opposite ends of the trunk, as in the higher articulata. The *ascaris*, like the other nematoid worms, has a single oral aperture at the anterior extremity of the body; the three marginal lobes of the mouth are provided with minute teeth and moved by distinct muscles, so that the mouth somewhat resembles that of the leech in its masticating apparatus. The œsophagus forms a wide elongated muscular sac, like that seen in the *halithea* and some other annelides; it is contracted at its lower part, and opens into a straight and wide intestinal canal with thin parietes, and where the limits of the stomach are seldom indicated by an inferior constriction.

B. We have arrived at an intestinal tube, prolonged to a greater or a less length, and provided with an aperture at either end, adapted for the respective purposes of receiving or of discharging the food of the animal. The next step towards perfecting the digestive apparatus is to dilate the tube in certain situations, in order that its contents may be allowed to rest in it, and to provide it with secreting appendages which may pour upon those contents such secretions as are adapted to act on it chemically or vitally. All this we see effected in the class of animals before us—a class which presents, we might say, *in petto*, almost all the characteristics of the very highest.

In the *diglena lacustris*, one of the *rotifera*, the sharp-pointed maxillæ and their muscular apparatus are succeeded by a lengthened and narrow œsophagus which opens into a short defined globular stomach. From different parts of the stomach two large and five small elongated cœca arise, which appear like hepatic or glandular follicles, and do not admit into their interior the larger undivided portions of the food contained in the general gastric cavity. From the pylorus, the intestine continues downwards narrow and nearly straight, to terminate in the cloacal opening at the posterior end of the body, close to the two fleshy peduncles.

C. We have now but to render the dilatations of the intestinal tube more definite—the secreting follicles more perfect—and to provide a triturating apparatus, and we have arrived at the essential items of the digestive organs of the animals highest in the scale. All this we are able to distinguish in the *insecta*.

"The digestive organs," says Dr. Grant, "have arrived at a high degree of development in insects, and already present, in an embryo-state, almost all the assistant chylopoietic organs of the highest animals, as the liver, the salivary glands, the pancreas, and many other parts important in the process of assimilation. They vary much, however, in their form and extent of development according to the consistence and the nutritious quality of the food, the peculiar living habits of the species, and the condition of the animals with regard to their metamorphosis. The mandibulate forms of the masticating organs are best adapted for comminuting hard substances, and the tubular form or proboscis for sucking food in a soft or fluid state, but even suctorial insects require some form of these hard parts to pierce the surface from which they are to obtain their liquid food. The mouth of insects is furnished with an upper and lower lip (*labrum* and *labium*), a pair of strong proximate *mandibles* and a pair of exterior *maxillæ* which move transversely. The labium and the maxillæ support each a pair of *palpi;* the dense posterior part of this lower lip forms the *mentum*, and its soft anterior portion supports the fleshy prominent *tongue*. The masticating organs present infinite varieties of form according to the difference of food in insects, as in other classes of animals, but the same constituent parts of the mouth can be recognized in all the different forms of mandibular and haustellar apparatus. The same buccal organs form broad, short, and strong cutting instruments, which move transversely, in those insects which subsist on hard food, and a long, slender, tubular apparatus, capable of extension and retraction, in those which suck fluid or soft substances. These parts often change from the one form to the other in the same insect, while it changes its kind of food in the progress of its metamorphosis; and where the food is the same in the larva and imago, the masticating organs preserve the same form in these two conditions of the insect. The food reduced by the mandibles and maxilla and mixed with the secretion of one or more pairs of salivary glands, is transmitted by a pharynx of variable length, to the œsophagus and alimentary canal. The œsophagus commences by a narrow canal which generally forms an enlargement of crop at its lower part, for receiving and collecting the food when first swallowed; this enlargement of the œsophagus is often covered with minute short glandular follicles which open into its interior. Below the crop is a small but strong muscular *gizzard*, with thick parietes, and provided internally with numerous longitudinal rows of hard sharp conical horny teeth. This muscular triturating stomach is most developed where the hardness of the food most requires its aid, as in most of the orthopterous and coleopterous insects; but where the food is liquid, as in most of the sucking hemiptera, the gizzard is scarcely perceptible. The largest, the most constant, and the most important gastric cavity in insects, is the long, wide and highly glandular *chylific stomach* which extends generally from the gizzard to the insertion of the hepatic ducts. The chylific stomach is, for the most part, amply furnished with considerable glandular follicles, which are developed from its whole parietes, and open by separate orifices into its interior. This cavity is frequently of great length, and partially divided by numerous transverse constrictions, it is then most wide and glandular at its anterior or proximal part, becoming narrower like the intestine at its lower portion. The intestine, from the termination of the chylific stomach to the anus, is most variable in its length and capacity, and in the number and extent of its dilatations. Like the masticating organs, the gastric cavities and the whole alimentary canal have their forms regulated and impressed by the kind of food which they are destined to assimilate, or the quantity they are adapted to consume. In the voracious and inactive condition of the developing larva, the stomach is often found of enormous capacity compared with the diminutive size to which it is reduced in the more parsimonious and active state of the mature winged imago.

The alimentary canal of insects presents a distinct internal mucous lining, an external peritoneal coat, and muscular fibres, both transverse and longitudinal, can be easily perceived in its parietes. The interior of the mucous coat presents a smooth surface, as in most of the lower invertebrata, having neither plicæ, nor valvulæ, nor villi, to increase its extent, and exterior to this there is commonly a loose cellular or follicular enveloping tissue. The exterior peritoneal coat forms a distinct thin mesentery, which is covered with the minute ramifications of tracheæ, and which connects the convolutions of the intestine with the interior of the abdominal segments. The ramifications of these white opaque air-vessels on the mesentery are seen in the common blue fly, and in most of the larger insects, without the aid of a lens, and appear like the branches of blood-vessels. The peristaltic motion of the intestine is obvious on opening the abdomen of the living insect; and in the short trunks of many of these animals, the intestine measures several times the length of the whole body." 349.

D. We have arrived at what we may consider a pretty perfect formula for a digestive apparatus. That consists of several subordinate apparatus—of one for trituration—for dilation or insalivation—for transmission (a pharynx or œsophagus)—for chymification and chylification, a stomach and intestinal canal, with its secreting appendages, pancreas and liver or their analogues—and, finally, for defæcation. It is not consistent with our present object to enter fully on the examination of each part of this complicated and perfect apparatus. But we may observe that, in the animals which have it, it is modified according to the circumstances which affect them, and on those modifications we shall lightly touch.

### C. MODIFICATIONS OF THE DIGESTIVE ORGANS IN THE HIGHER ANIMALS.

A. The circumstance which above all others modifies the digestive apparatus, is the animal or vegetable nature of the food. We see this as strongly marked in the articulata as in the vertebrata, and hence the great division of animals into carnivorous and granivorous.

In the *carnivorous*, the digestive tube is shorter—the glandular organs frequently less perfect—the triturating infinitely less so—the intestinal dilatations less marked and less disposed to affect the form of cœca.

In the *granivora*, the converse of all this is seen, and the triturating apparatus especially, perfected.

We may compare, for example, the carnivorous insect—the *cicindela campestris*, with a herbivorous—the common *coleopterous cockchaffer*.

In the *former*, the digestive canal passes nearly straight through the body. The œsophagus, commencing narrow as usual from the posterior opening of the head, dilates below into a wide crop, presenting several longitudinal rows of very minute follicles. The crop is succeeded by a short muscular gizzard, and this by a capacious chylific stomach, covered with numerous glandular follicles, and tapering downwards to its pyloric extremity, where it is perforated on each side by two simple convoluted hepatic vessels. From the chylific stomach the intestine continues downwards very narrow, and nearly straight, to a short dilated colon, which contracts before it terminates in the cloaca.

In the *cockchaffer*, which feeds on the leaves and shoots of our garden plants, the whole digestive apparatus is long, complicated, and capacious. The œsophagus passes out narrow from the head, and dilates below into a short conical crop, which is succeeded by a very minute gizzard, and a long convoluted chylific stomach. The anterior portion of this lengthened glandular stomach is wide and sacculated by numerous transverse strictures, and terminates insensibly in a narrow convoluted pyloric part, which dilates into a small round vesicle at the lower end, where it receives the openings of the hepatic vessels. The two hepatic vessels on each side are here, in accordance with the coarse nature of the vegetable food, very long, wide, and convoluted, and have their secreting surface greatly extended by the development of innumerable small lateral follicles, which give them a pinnated form throughout the greater part of their course; thus presenting the most complicated condition of the liver met with in insects. The lower portion of the intestinal canal has also its capacity increased by distinct dilatations on the parts analogous to the colon and the rectum.

B. The varieties exhibited by one of the dilatations of the digestive tube—the stomach, display the modifications dependent on circumstances in a striking manner.

In a pure carnivorous mammal it may be a simple globular sac. From that simple form we may trace it to the less carnivorous tribes, to the quadrumana and man, in whom it has become transversely elongated with a cardiac fundus or cæcum. In many of the rodentia, the thin membranous cardiac portion forms a considerable cæcum, and is partially separated by a constriction from the more muscular pyloric half of the cavity. The great development of the gastric glands around the cardiac orifice of the stomach in the beaver and the wombat, is required by the coarse vegetable food on which they subsist, and points out an analogy between this part and the glandular infundibulum at the cardiac orifice of the gizzard in birds. In several phytophagous mammalia, belonging to different orders, as among the pachydermata, masupialia, edentata, and even quadrumana, internal folds or external cœca divide the cavity of the stomach to a greater or less extent, and from the epidermic lining extending over the cardiac sacs thus formed, we observe a gradual transition to the complex stomachs of the cetacea and ruminantia, where the several compartments have different structures and functions.

But it is in the ruminantia that the stomach has attained its acmé of development. As Dr. Prout has observed no cookery can enable man to subsist upon the grasses. Yet they furnish the food of a most important class of animals, and these being chiefly the food of man himself, he thus indirectly lives upon the grasses. The means by which the ruminantia convert the grasses into food are pretty clearly described by Dr. Grant.

"The ruminating quadrupeds have the masticating and salivary organs most perfect, the œsophagus long and narrow, four gastric cavities with distinct functions, the intestine and colon long, capacious and distinct, and an enormous *cæcum coli*. The œsophagus, as seen in the annexed figure of the stomach of the *lama* of Peru, enters directly into the first large cavity or paunch (*ingluvies*), placed on the left side, analogous to the crop of birds, and serving the office of the cheek-pouches of other quadrupeds. This capacious cavity is partially subdivided by large internal folds, and is lined with closs, small but lengthened villi, covered with a thick epidermis. The same epidermic covering is continued distinctly over the second and third cavities. The second stomach or *reticulum* placed more anteriorly and to the right of the former, is much smaller, and has its mucous lining elevated into reticulate folds forming polygonal shallow cells. This cavity also communicates with the œsophagus, and presents two thick muscular valvular folds across the opening of communication, by the closing of which a canal is formed which leads to the third cavity of the stomach.

The paunch receives the crude-unmasticated vegetable food collected in large quantity while the animal is erect and grazing, and the process of rumination generally commences when this cavity is filled, and the animal is reclining at rest. In rumination, small portions of the unmasticated food, moistened and softened in the paunch, pass into the second cavity to be sent by its contraction, as a bolus, upwards through the muscular œsophagus to the mouth. After being thoroughly masticated and salivated in the mouth, the bolus returns, as a soft pulp, by the œsophagus; and, its stimulating quality being now altered, it finds the two valvular folds at the lower end of the œsophagus closed, and shortened by contraction, and is directed by the short canal they thus form, into the third, and thence into the fourth cavity of the stomach. The third or foliated stomach (*omasum*) is generally the shortest and smallest, though elongated and narrow in the *camelus* and *auchenia*, and is provided internally with numerous longitudinal, alternately small and large folds, having their free margins directed to the centre of the cavity. The second and third cavities have their mucous membrane covered with small villi and distinct epithelium, like those lining the first stomach. The third cavity leads directly to the fourth or

*abomasum,* which is next in capacity to the paunch, lined with a soft highly vascular mucous coat, provided internally with large longitudinal folds, and apparently destitute of epidermic lining. The structure and form, and secretions of this fourth cavity, placed on the right side of the others, render it the proper digestive stomach, and the most analogous to the single digestive sac of carnivorous and higher quadrupeds." 411.

The means by which the camel is enabled to carry a supply of water sufficient for its consumption in the desert have been fully described by Sir Everard Home. The following brief notice of the apparatus by Dr. Grant will be sufficient to give our readers an idea of it.

In the camels, dromedaries and lamas, numerous rows of large, quadrangular, deep water-cells are developed on the parietes of the second stomach, and on the parts of the paunch next to that cavity. These cells are surrounded by muscular fibres which, by their contraction, are capable of excluding the food from the water-cavities; and by the gradual opening of the cells, the water is allowed to mix in successive small portions with the digesting aliment. These animals are thus enabled to convey and economise a large supply of pure water, received at long intervals in the arid plains they inhabit. The second stomach is more appropriated to the retention of water than the large paunch, and receives it directly from the mouth, unmixed with the food, pouring over to the cells of the first stomach a quantity of the fluid when its own are filled.

It is curious to observe the manner in which Nature not unfrequently accommodates herself to circumstances, and makes the same organs with a slight alteration perform almost different offices. The transformations in the embryo and fœtus, the metamorphoses of insects, will at once present themselves to the mind of the anatomist, and he finds in the ruminantia an instance of the same description. Their complicated stomachs are calculated and intended for the digestion of most indigestible substances. But the ruminating animal is a calf before it is a bull, and, when a calf, lives on its mother's milk, a highly nutritious liquid. Observe the modification in the action of the stomachs at this period.

" The fourth stomach of the ruminantia is the first developed, the largest, and alone employed for digestion, during the earlier periods of existence and during lactation. The milk, in suckling, passes down through the œsophagus to the closed valvular folds, which check its entrance into the first or second stomach, and convey it along the canal which they form, directly to the third or foliated cavity. The third stomach not having been yet distended with solid food so as to separate from each other its numerous contiguous laminæ, it merely forms a tube through which the milk passes into the fourth stomach; and thus the nutritious fluid of the parent is conveyed directly into the proper digestive stomach of the suckling ruminant, without being accumulated or retarded in any of the previous cavities." 412.

c. The triturating apparatus presents in the different classes of animals a remarkable variety. In the highest class we see it principally at the very entrance of the alimentary canal, and the moveable jaw, with its teeth adapted for cutting and for grinding, is of course familiar to us all. But in many animals the triturating implements are not at the entrance of the digestive tube, but at the stomachic dilatation. We see this not unfrequently in the articulata.

The small mouth, says Dr. Grant, of the *halithea aculeata*, furnished with two conical tentacula or antennæ, opens into a short membranous œsophagus, which terminates in a large muscular stomach with thick firm parietes and a strong coriaceous lining. The entrance of this muscular cavity is furnished with four sharp, triangular, converging, horny teeth analogous to those of the gastric toothed cavity of insects and crustacea; and this sac is also analogous to the muscular stomach of the *arenicola*, *lumbricus*, and many other annelides, and to that of the *ascaris* and other nematoid entozoa.

In the crustacea, the gastric teeth are seen in a state of high development. The wide buccal cavity of the decapods, surrounded with complicated organs of sense and of mastication, opens by a very short and narrow œsophagus into a capacious stomach, provided internally with several pairs of solid calcareous teeth, and occupying the anterior part of the cephalo-thorax. As their watery element almost bathes this gastric cavity, they require no salivary glands to soften their moist food. The gastric teeth, colored and shed like the exterior shell, are symmetrically disposed near the pylorus, and are supported by thin elastic calcareous laminæ, to which powerful muscles are attached, and which cause the teeth to meet with precision in grinding the contents of the stomach.

Another form of gastric triturating apparatus is the gizzard. Existing in the articulata, it attains its maximum in birds. It is a small muscular stomach, having a horny epidermic lining, and, to assist in the comminution of food, the bird swallows stones, which, acted on by the muscular parietes of the gizzard, bruise and grind the grain. Oddly enough, Spallanzani contended that this was from stupidity. The stupidity was Spallanzani's. Dr. Grant's description of a gizzard is as follows:—

"These muscular parietes of the gizzard form two strong digastric muscles, one anterior, the other posterior, with white shining tendons, in most gallinaceous and granivorous birds, and in many aquatic and other species. In most rapacious and carnivorous birds, the parietes of all the three gastric cavities are thin and highly extensible, and form almost one continuous stomach, with slight constrictions between its parts. The thin membranous gizzard however of these birds presents a distinct anterior and posterior central tendon, analogous to those of the two ordinary digastric muscles, and from which the muscular fasciculi, more or less developed in different species, radiate to the margins of the cavity. When this third gastric cavity is provided with strong muscular parietes, as in the gallinaceous birds, its internal epidermic lining forms a thick coriaceous dense coat, to protect the soft parts from laceration, and to enable them to act with effect upon their heterogenous contents. From the want of teeth in the mouth to act upon their hard food, these granivorous birds convey pebbles and other dense substances into their gizzard, to reduce their food, like the gastric teeth of crustacea, insects, many gasteropods, and other invertebrata; but in the carnivorous birds, with a thin membranous gizzard, no pebbles are swallowed or required, the activity of the secretions affecting all the necessary changes in the conditions of the food, aided by the high temperature, and the movements of the canal. From the proximity of the cardiac and pyloric orifices of the gizzard, it forms a sac open only above, and is not provided with the pyloric sphincter muscle, so common in other vertebrated classes. This free and wide pyloric orifice allows the food, partially digested in the three gastric cavities, and reduced by the muscular action of the gizzard, to pass out, in small successive portions, to the commencement of the duodenum. The small internal capacity of this strong grinding organ, which is chiefly filled with pebbles, necessitates the development of other gastric cavities between it and the mouth, to receive a sufficient quantity of coarse vegetable food for the maintenance of these large and heavy birds." 403.

The powers of the gizzard were put to the test by Felix Plater. He gave a hen a Louis d'or—a royal meal. In a short time, we forget just now how long, it lost sixteen grains of its weight. An onyx lost in the gizzard a fourth of its weight.

Carnivorous birds, who have a very imperfect gizzard, may be brought to live on grain, when a powerful gizzard becomes developed. On the other hand, granivorous birds, who swallow stones, cease to do so if brought to feed on animal matters.

D. The varieties of *beak* in *birds* are highly interesting. They represent the teeth in being organs of offence or defence, the lips, &c. in being organs of prehension. Dr. Grant's remarks on this head are succinct, but not uninteresting.

The jaws, he says, have their alveolar margins covered, like those of chelonia, with horny plates, which vary in their forms, according to the kind of food, like the teeth of mammalia. The nearest approach to the teeth of quadrupeds is seen in the thin horny laminæ disposed along the sides of the bills in the *mallard*, and some other aquatic birds; and in the earliest condition of the birds the laminæ begin by a series of small detached tubercles, provided each with its pulp, its nerve, and its vessels, like the horny maxillary plates of the whale, and the more solid calcareous teeth of other vertebrated animals. The broad depressed bills of ducks, geese, swans, and many other aquatic birds, with dentated edges, and soft sensitive lips, are well adapted for obtaining worms or other small objects under water or in mud, and they commonly present a well-marked dental distribution of the alveolar nerves and blood-vessels, as well as a high development of the second and third branches of the trigeminal nerves. The flat spatulate jaws of the spoon-bills are adapted for quick lateral motion in the waters, and for extracting minute animals from the moist banks of lakes and rivers. The submaxillary pouch of the pelican serves as a net for seizing fishes; the straight sharp bills of cranes and storks dart with precision through the water upon their moving prey, and the long compressed bills of cormorants, gulls, albatroses, and many predaceous aquatic birds, terminate above in a sharp inverted hook, to seize firmly the smooth scaly bodies of fishes. The broad bills, with cutting edges, of the strutheous birds, are adapted to prune the leaves and shoots of plants, and the long narrow bills of woodpeckers to be inserted into small crevices to seize minute insects; and most of the insectivorous order of birds have a similar structure on a smaller scale. The long tubular beak of the humming birds is suited for insertion into the corollæ of flowers. In the grosbeaks and crossbills, the sparrows and buntings, and all the granivorous order, and in the larger gallinaceous birds, the bills form stronger and shorter cones, broader at the base, to break down and remove the hard coverings of grains. In the climbing frugivorous cockatoos, parrots, and maccaws, the broad and powerful bills serve as prehensile organs, and to break the hard shelly coverings of seeds. The bills of eagles and vultures, hawks and owls, and other rapacious birds, are strong, short, compressed, arched, curved at the point, dense in their texture, and with sharp cutting edges, to seize, and tear, and cut the flesh of living prey. So that the forms of these external parts correspond with and indicate the structure of the internal organs of digestion, and afford useful zoological characters for the divisions of this class.

E. The length to which this article has already run precludes the idea of our proceeding much farther. We are tempted to allude to one, and but one, other subject. Every one is aware how feeble, in point of chemical action, the salivary secretion is. Yet in certain circumstances, this same secretion becomes a most formidable instrument of death in the jaws of the creature that possesses it. The parotid of the rattle-snake is the poison gland. That gland lies behind and below the orbit on each side, contained in a wide cavity, is embraced by a slip of the temporal muscle, and sends its long duct to the perforated base of the poison fang.

Here, then, we pause. In succeeding numbers we shall resume the fascinating subject of Comparative Anatomy, and present a few sketches of the development of the various apparatus by which the vital functions are performed in the ascending scale of animals.

We recommend the Outlines of Dr. Grant to the attention of our readers, and particularly of the younger portion of them. They will find much delightful and some useful knowledge in our author's pages. The work, from its low price, *may* be in the hands of most men—*should* be in the hands of all.

